# Compression Isotherms of Monomolecular Films of 7β-alkyl Cholic Acid Derivatives on an Acidic Aqueous Substrate and Their Interactions with Lecithin Reveal the Conformations of the Alkyl Chain and Steroid Skeleton

**DOI:** 10.3390/ijms26125760

**Published:** 2025-06-16

**Authors:** Dileep Kumar, Mihalj Poša

**Affiliations:** 1 Laboratory for Chemical Computation and Modeling, Institute for Computational Science and Artificial Intelligence, Van Lang University, Ho Chi Minh City 70000, Vietnam; 2 Faculty of Applied Technology, School of Technology, Van Lang University, Ho Chi Minh City 70000, Vietnam; 3 Department of Pharmacy, Faculty of Medicine, University of Novi Sad, Hajduk Veljka 3, 21000 Novi Sad, Serbia

**Keywords:** bile acids, steroid skeleton, conformational analysis, monomolecular film, molecular associations

## Abstract

This study analyzed the compression isotherms of 7β-alkyl cholic acid derivatives and compared them to those of cholic and deoxycholic acids to elucidate their orientation and molecular interactions (acidic aqueous substrate—pH 2; NaCl concentration—3 M; temperature—*T* = 298.15 K). It was found that the compression isotherm of the 7β-octyl derivative of cholic acid in the monomolecular layer is most similar to the compression isotherm of deoxycholic acid. In 7β-alkyl derivatives of cholic acid, the hydrophobic interaction energy in their aggregates from a monomolecular film increased with the length of the alkyl chain. However, this energy did not increase linearly with C atoms, suggesting the existence of a conformational equilibrium. In binary mixtures of the tested bile acids and lecithin, only the 7β-octyl derivatives of cholic acid and deoxycholic acid had orientations in which the steroid skeleton had a “vertical” position, i.e., only the C3 OH group was immersed in the aqueous substrate, which resulted in the maximum hydrophobic interaction with lecithin. In 7β-octyl derivatives, part of the octyl chain probably also participated in the interaction with lecithin. In 7β-propyl and 7β-butyl derivatives, C7 alkyl groups sterically shielded the C7 α-axial OH group. However, in the 7β-ethyl derivative the C7 OH group was not additionally sterically shielded, so this derivative, similarly to cholic acid, partially dissolved in the aqueous substrate after the collapse point.

## 1. Introduction

**Figure 1:**
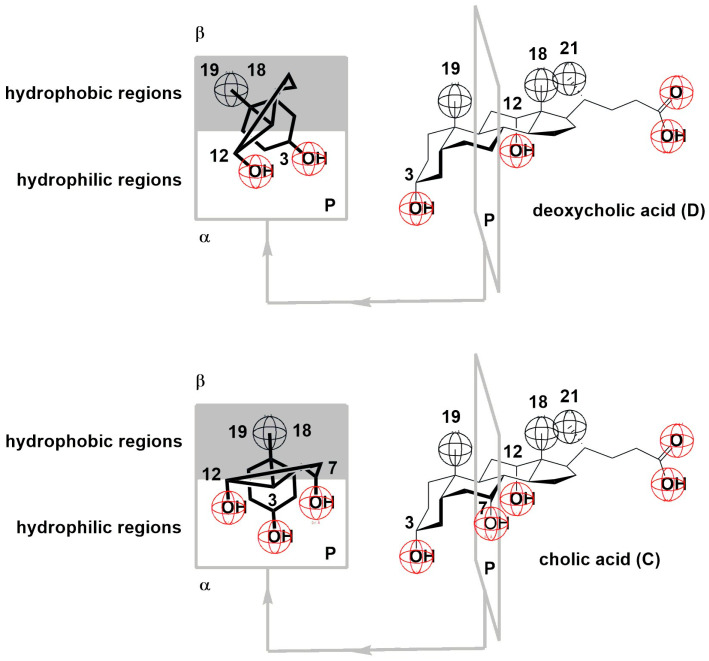
The ratio of hydrophobic and hydrophilic regions in deoxycholic and cholic acids. The specific geometry, i.e., parallel C-OH bonds of equatorial (C3) and axial (C7 and C12) OH groups, is a consequence of the cis connection of the A (first) and B (second) rings of the steroid skeleton. Therefore, the A ring with axial bonds rotates by 60° relative to the axial bonds of the B ring, which results in the C3 equatorial OH group becoming pseudoaxial (if viewed in isolation, the A ring C3 OH group has an equatorial orientation; however, in the steroid skeleton, it is parallel to the axial OH groups of the B and C rings, and therefore, it is called pseudoaxial). P—projection plane into which all cross sections of the steroid skeleton are mapped.

Bile acids have a unique structure characterized by a rigid cyclopentanoperhydrophenanthrene ring featuring a hydrophobic convex surface (β side) and a hydrophilic concave surface (α side) (Figure 1). This structure makes bile acids a fascinating subject of study [1,2,3,4]. Bile acid anions can form small, primary micelles in aqueous solutions, as described by the Small–Kawamura concept. In these primary micelles, the steroid skeletons of the bile acids are in mutual contact across the molecule’s β side, primarily due to hydrophobic interactions [5,6,7,8,9,10,11,12]. At higher concentrations, hydrogen bonding leads to larger secondary micelles [13,14,15,16,17,18].

Bile acid salts (steroid biosurfactants) are produced in vertebrates’ livers, primarily in the form of cholic and chenodeoxycholic acids. Intestinal bacteria modify these primary bile acids into secondary ones, such as lithocholic and deoxycholic acids. When mixed with phospholipids, bile acid anions form mixed micelles that solubilize cholesterol in the bile canaliculus and play a crucial role in emulsifying and solubilizing lipids, fat-soluble vitamins, and drugs in the small intestine [3,19,20].

While the micellar form of bile acid salts is highly beneficial in pharmaceutical formulations as it enhances the solubility of hydrophobic drugs, it is important to recognize potential drawbacks [21,22,23,24,25,26,27]. Its detergent properties can alter cell membrane permeability, facilitating drug transport [28,29,30]. However, this can also increase membrane toxicity and the hemolytic potential of hydrophobic bile acid anions, underscoring the need for caution in their use [31,32].

Studying the properties and characteristics of insoluble and soluble monolayers provides valuable insights into molecular dimensions, intermolecular interactions, and interactions with substrates (the substrate carries a monomolecular layer). In the Langmuir trough, a movable barrier can decrease the surface area, allowing for measurement of the pressure exerted by the film. This process generates a compression isotherm that illuminates the relationship between the average surface area per molecule and the surface pressure [13,33,34,35,36].

Two methods are commonly used to study molecular interactions in mixed films. The first method involves creating binary mixtures of two insoluble components, such as bile acids, and biologically relevant compounds, like phospholipids, triglycerides with fatty acids, and cholesterol. These mixtures are then spread on the substrate’s surface to obtain compression isotherms. The second method involves spreading an insoluble component, such as phospholipids, on the substrate. Next, a soluble substance, like bile acid salts, is injected into the aqueous phase. After allowing sufficient time for the soluble substance to equilibrate with the surface, the isotherm is obtained [15,29,37,38].

**Figure 2:**
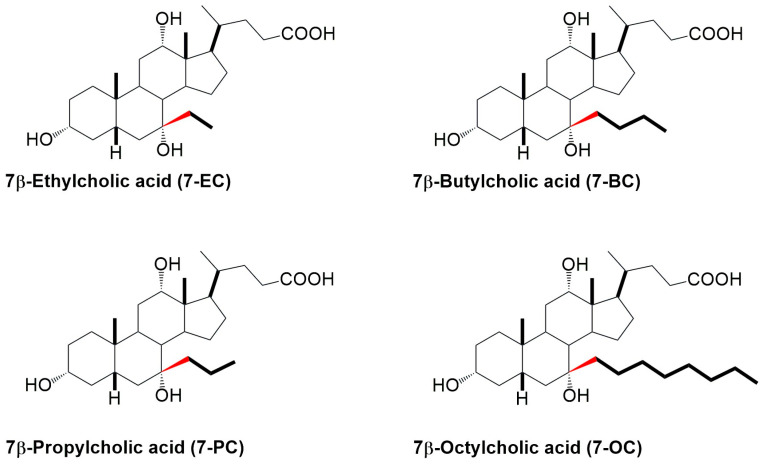
The studied 7β-alkyl derivatives of cholic acid.

When using bile acid salts in pharmaceutical formulations, the goal is usually to have as large a hydrophobic surface as possible (with an increase in the hydrophobic surface, its capacity for micellar solubilization of the hydrophobic molecular guest increases) but not to lose its ability to form micelles, so that the membranolytic ability is not greater than that of the sodium salt of deoxycholic acid. Therefore, 7β-alkyl derivatives of cholic acid were synthesized (Figure 2) and Na salts were investigated in micellization thermodynamics [39,40]. It was found that the change in the heat capacity of demicellization to 7β-butyl derivatives (anions) increases linearly with the C atoms of the C7 alkyl group. In contrast, after that, it increases more slowly (not linearly). In the anion of the 7β-octyl derivative of cholic acid, the C7 alkyl group, instead of taking an antiperiplanar conformation, took a conformation in which it bent toward the angular methyl groups of the steroid skeleton [40].

The aim of this study was to evaluate how the length of the 7β-alkyl chain in cholic acid derivatives affects their molecular orientation, packing behavior in monolayers, and interaction with lecithin (Figure 2). The question we asked was whether, above a certain length, the C7 alkyl chain imparts some new properties to the molecule in terms of compression isotherms and interactions with lecithin in the monomolecular layer that do not exist in the basic molecule (cholic acid) and shorter 7β-alkyl derivatives, such as ethyl and propyl derivatives. The hypothesis was that in longer alkyl chains, the beginning of the alkyl segment has the same conformation as in 7β-ethyl and 7β-propyl derivatives—an antiperiplanar conformation to prevent steric strain with the B and D rings of the steroid skeleton. With the extension of the alkyne chain, more twisted and synclinal conformations are possible, which can be directed toward the angular methyl groups of the steroid skeleton without the formation of added steric strain.

## 2. Results

**Figure 3:**
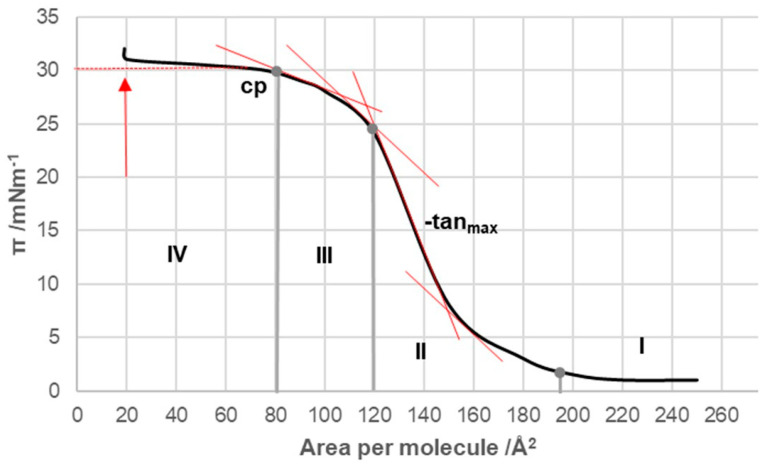
Compression isotherm of deoxycholic acid at 298.15 K on a substrate of an acidic aqueous solution with pH 2 and an NaCl concentration of 3 M; cp—collapse point of surface film; tan_max_—tangent with the maximum negative slope: tan_max_ < 0 and −tan_max_ > 0.

The compression isotherms of the tested bile acids (Figure 1 and Figure 2) were measured at 298.15 K on a substrate consisting of an aqueous solution with a pH of 2 and an NaCl concentration of 3 M. For deoxycholic acid, four distinct regions (I–IV) could be identified in the compression isotherm (Figure 3). In the first region, according to Crip’s equation, the number of degrees of freedom, *F*, is defined as follows [13]:

(1) $$F=C-P_{b}-(q-1)$$

In this equation, *C* represents the number of components in the system, *P_b_* corresponds to the number of macroscopic phases, and *q* denotes the number of phases in the surface monomolecular film. In region I of the compression isotherm, the surface pressure can be considered constant, which means *F* = 0. As the system had two components, the aqueous substrate and deoxycholic acid from the surface film, and one macroscopic phase (aqueous solution), two surface phases were needed (*q* = 2) for the condition *F* = 0 in region I of the compression isotherm. One surface phase was analogous to the vapor phase, which was in equilibrium with the other surface phase—the surface liquid phase, i.e., the liquid-expanded state (the phenomenon is analogous to the condensation of the vapor phase at constant temperature, while the volume of the system decreases and condensation continues until the pressure is constant). Similarly, in region I, while the surface area decreased, and, at the same time, there was a surface phase analogous to the vapor and liquid phases, the surface pressure was constant until then.

In region II of the compression isotherm (Figure 3), the total amount of deoxycholic acid was in a quasi-liquid state, i.e., *q* = 1; therefore, *F* = 1, the surface pressure (π) increased with the decreasing surface area per molecule and the surface phase, which was analogous to the 3D liquid phase, was compressed. In region III of the compression isotherm, *F* = 1 was still present. However, there was a rearrangement in the packing of molecular aggregates in the surface monolayer—a liquid-condensed phase was obtained. In area IV, the macroscopic solid phase of deoxycholic acid appeared so that there were two macroscopic phases (the other was an aqueous solution) and one phase in the surface monomolecular layer—a quasi-liquid phase with close packing; therefore, according to Equation (1), *F* = 0, i.e., with a decrease in the surface area per molecule (increase in surface pressure), deoxycholic acid molecules were pushed out of the surface monomolecular layer into the solid insoluble macroscopic phase of the same molecule. The arrow in Figure 3 indicates the point in the compression isotherm when the total amount of deoxycholic acid was present as a macroscopic solid phase. In bile acids, the point of collapse is defined as the point in the compression isotherm corresponding to the transition from area III to area IV. At the point of collapse, the surface occupied by one molecule of deoxycholic acid was 80 Å^2^ (Figure 3), while the collapse pressure was 30 mNm^−1^, corresponding to the values found in the literature [13].

**Figure 4:**
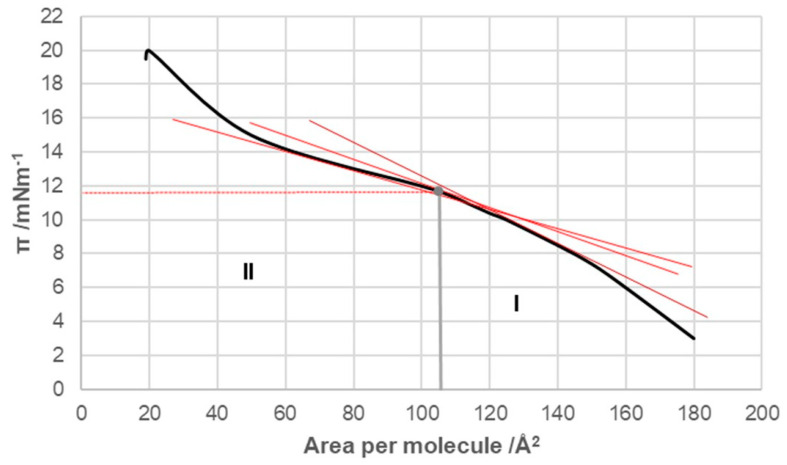
Compression isotherm of cholic acid at 298.15 K on the substrate of an acidic aqueous solution with pH 2 and an NaCl concentration of 3 M.

With cholic acid, two regions were observed in the compression isotherm (Figure 4): region I corresponded to regions II and III of the compression isotherm of deoxycholic acid (Figure 3), while region II corresponded to region IV. Namely, after the collapse point, with a further increase in surface pressure, not only was cholic acid squeezed out of the surface film into the undissolved macroscopic phase but its solubility in the aqueous solution of the substrate also increased at the same time. The collapse point of cholic acid was covered by an area of 108 Å^2^/molecule (literature value 105 Å^2^/molecule [13]), which is larger than the same area for deoxycholic acid. Namely, cholic acid at the air–water phase interface was immersed in the aqueous solution with three steroid OH groups (hydrophilic concave surface) so that the entire α side of the steroid skeleton was next to the surface of the aqueous substrate. In contrast, deoxycholic acid was immersed in the aqueous phase of the substrate with two steroid OH groups, forming a hydrophilic edge on the concave surface of the steroid skeleton, i.e., deoxycholic acid covered a smaller surface of the aqueous substrate (Figure 1, projection plane P). The collapsing surface pressure of cholic acid was 11.8 mNm^−1^, while the value in the literature is 14 mNm^−1^ [13].

**Figure 5:**
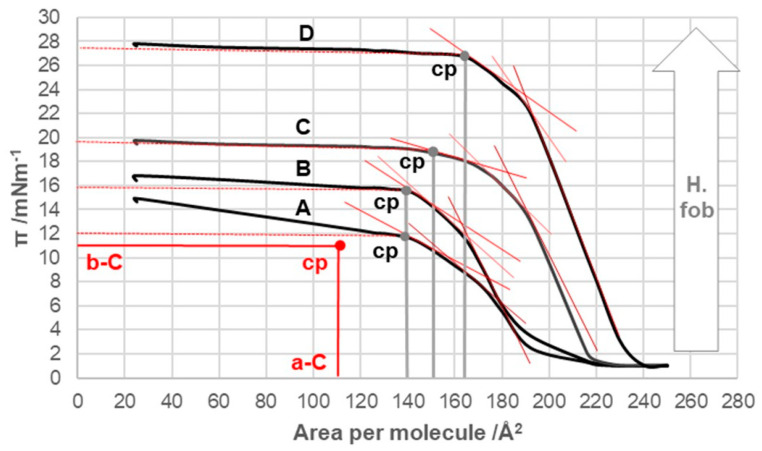
Compression isotherms for the examined 7β-alkyl derivatives of cholic acid (A = 7-EC, B = 7-PC, C = 7-BC, and D = 7-OC) at 298.15 K on the substrate of an acidic aqueous solution with pH 2 and an NaCl concentration of 3 M. cp—collapse point of the surface film; b-C and a-C determine the cp of cholic acid; H.fob—hydrophobicity.

In the 7β-alkyl derivative of cholic acid (Figure 2), the compression isotherm of the 7-EC derivative was most similar to the compression isotherm of cholic acid (Figure 4 and Figure 5). Namely, after the collapse point, with a decrease in the surface area per molecule (i.e., when the monomolecular layer was compacted), the surface pressure (π) increased slightly in the isotherm, indicating the solubility of the 7-EC derivative (similar to C, Figure 4) in the aqueous substrate.

With an increase in the number of carbon atoms in the C7 alkyl chain of the steroid skeleton, the compression isotherms shifted toward higher values of the surface pressure and the higher values of the surface occupied by one molecule (Figure 5). Therefore, with increasing hydrophobicity, the collapse points moved toward higher values of π (Table 1). However, the collapse pressure, even for 7-OC derivatives, did not reach the collapse surface pressure of deoxycholic acid. Starting from the 7-PC derivative, the compression isotherms were more similar in shape (flow) to those of deoxycholic acid than to those of cholic acid.

In bile acids C, 7-EC, 7-PC, 7-BC, and 7-OC (Figure 4 and Figure 5), the surface pressure of around 20 Å^2^/molecule dropped sharply in contrast to deoxycholic acid (Figure 3). This phenomenon occurred with some bile acid derivatives when the total amount of the densely packed surface liquid phase, due to the compression of the surface film, passed into a continuous solid phase, which, upon further compression, disintegrated and formed a multimolecular layer of the solid phase with different structural characteristics (the continuous solid phase lost its resistance to compression) [36,41].

At 5 mNm^−1^ surface pressure (substrate aqueous solution with pH 2, NaCl concentration 3 M), dependences of the average surface per molecule from the binary mixture of bile acids and lecithin were obtained based on the mole percentage (fraction) of lecithin.

**Figure 6:**
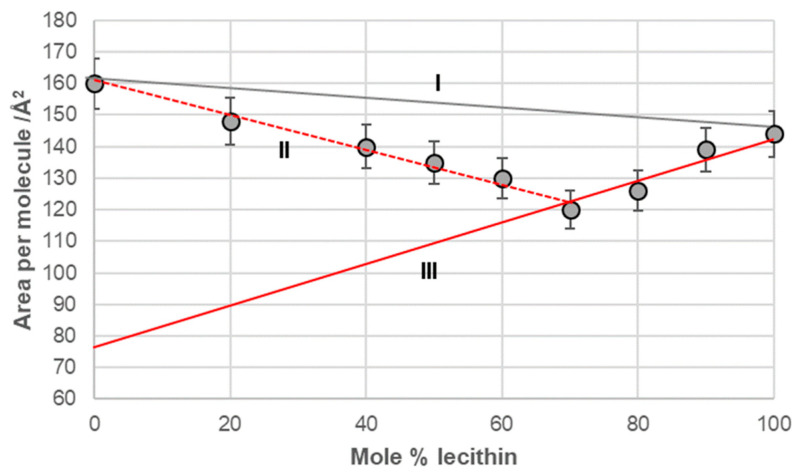
Average area per molecule of deoxycholic acid–lecithin mixtures at 5 mNm^−1^ pressure represented as mole percentage of lecithin (aqueous solution with pH 2 and an NaCl concentration of 3 M).

**Figure 7:**
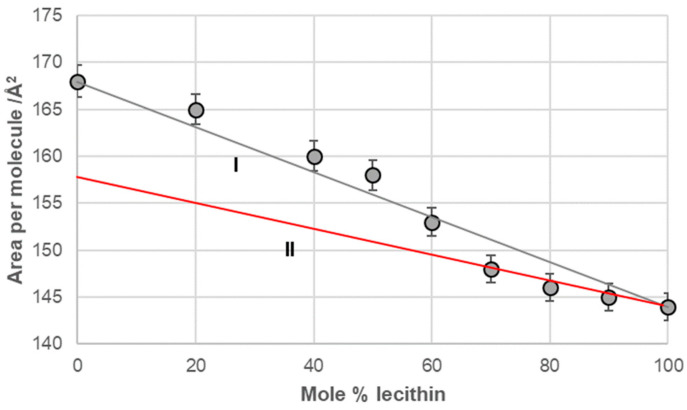
Average area per molecule of cholic acid–lecithin mixtures at 5 mNm^−1^ pressure represented as mole percentage of lecithin (aqueous solution with pH 2 and an NaCl concentration of 3 M).

**Figure 8:**
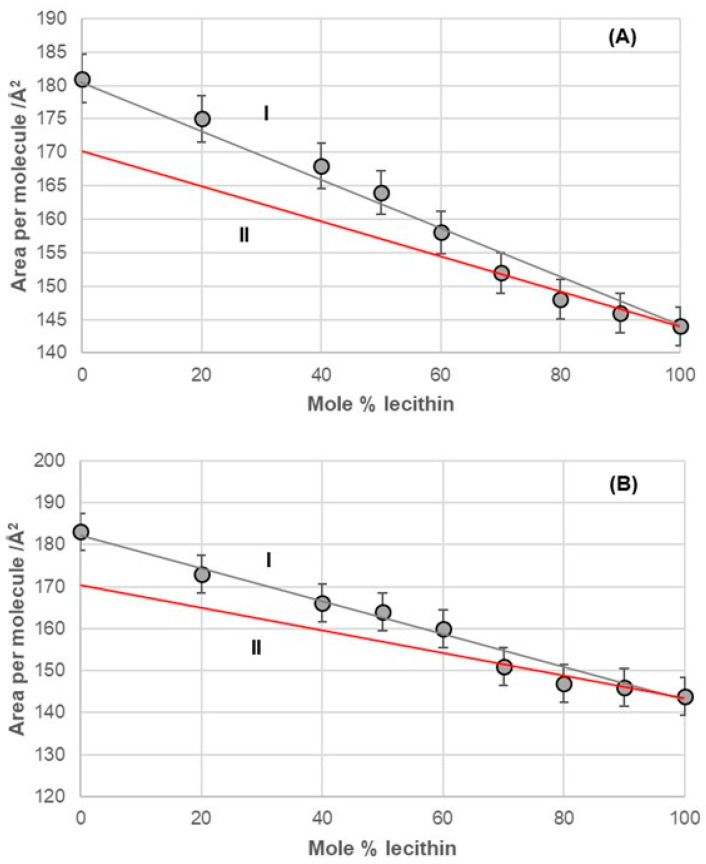
Average area per molecule of (**A**) 7-EC–lecithin and (**B**) 7-PC–lecithin mixtures at 5 mNm^−1^ pressure represented as mole percentage of lecithin (aqueous solution with pH 2 and an NaCl concentration of 3 M).

**Figure 9:**
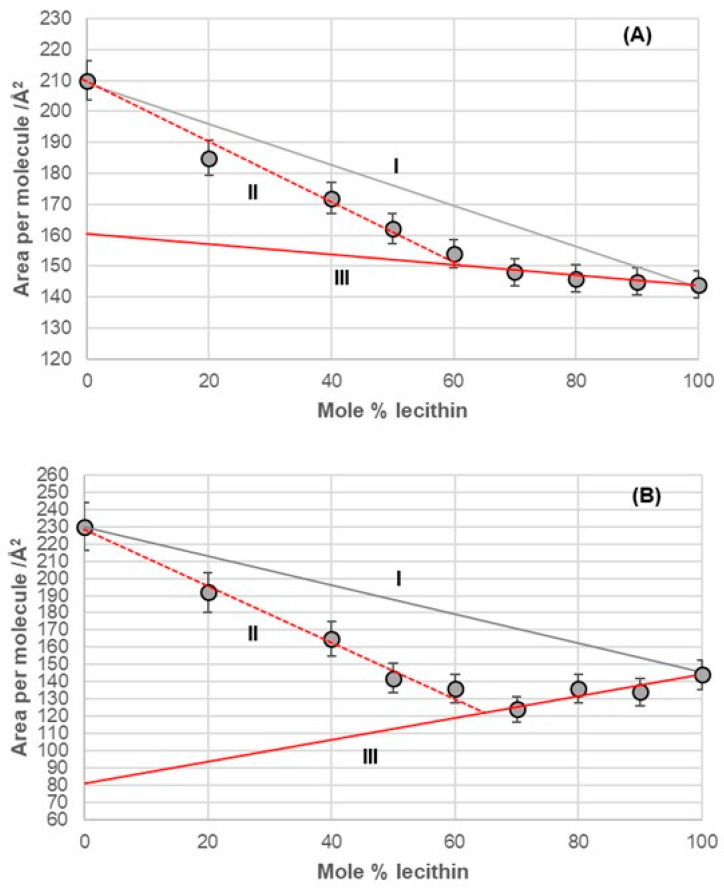
Average area per molecule of (**A**) 7-BC–lecithin and (**B**) 7-OC–lecithin mixtures at 5 mNm^−1^ pressure represented as mole percentage of lecithin (aqueous solution with pH 2 and an NaCl concentration of 3 M).

Line I in Figure 6, Figure 7, Figure 8 and Figure 9 represents a linear function (additivity line) according to which bile acids and lecithin would behave in a monomolecular film if they formed either an ideal binary mixture or two separate immiscible surface phases: bile acids and lecithin (separate islands of molecules). With deoxycholic acid, there was a negative deviation from line I (Figure 6), which means there are synergistic interactions between deoxycholic acid and lecithin in their binary mixture in a monomolecular film. Lines II and III (Figure 6) indicate that deoxycholic acid behaved differently in the investigated binary mixture when the mole percentage of lecithin was greater than 70%. The binary mixture of cholic acid and lecithin, with up to 60% of the mole percentage of lecithin, generally behaved according to the additive rule (line I, Figure 7), while at more than 60% of the mole percentage of lecithin, the binary mixture showed synergistic properties.

The 7-EC–lecithin and 7-PC–lecithin binary mixtures (Figure 8) displayed similar behavior to the cholic acid–lecithin mixture. Specifically, both mixtures generally followed the additive rule. However, at high mole percentages (fractions) of lecithin in these binary mixtures, they demonstrated synergistic properties relative to the hypothetical ideal state of the mixtures. In this context, the only observed change was in entropy due to the formation of the mixture within the monomolecular film.

The binary mixture of 7-BC and lecithin in the monomolecular layer demonstrated synergistic interactions between the alkyl derivatives of bile acids and lecithin across the entire range of lecithin mole percentages (fractions). However, this mixture exhibited different behavior in the monomolecular layer, depending on the concentration of lecithin. Specifically, from 0% to 60% of lecithin (represented by line II in Figure 9A), the packing in the 2D liquid phase differed from that with 60% to 100% of lecithin (represented by line III in Figure 9A). This behavior resembled that of the binary mixture of deoxycholic acid and lecithin (Figure 6). The key difference was that line III intersected the abscissa at different surface areas, indicating a distinct orientation for deoxycholic acid and 7-BC derivatives in their mixtures with lecithin.

The binary mixture of 7-OC and lecithin in a monomolecular film (Figure 9B) exhibited similar behavior to the binary mixture of deoxycholic acid and lecithin (Figure 6). This similarity was evident in the synergistic interactions observed throughout the entire mole percentage range of lecithin in the binary mixture and in the intersection of line III with the abscissa, which occurred at comparable molecular surface sizes for both mixtures. This indicated that bile acids display identical orientations in binary mixtures with a high proportion of lecithin, such as those containing deoxycholic acid and 7-OC derivatives. These orientations differed from those in the region associated with true II (refer to Figure 6 and Figure 9B).

## 3. Discussion

The resistance of monomolecular films to compression, often referred to as elasticity, is typically characterized by the compressibility modulus, denoted as

(2) $$K=-A{\left(\partial \pi /\partial A\right)}_{T}$$

In this equation, A represents the area per molecule, π is the surface pressure in the films, and *T* is the temperature. An increase in the compressibility modulus indicates a greater film resistance to compression and signifies a gradual transition of the film’s behavior from fluid-like to solid-like. Conversely, a decrease in the compressibility modulus indicates the opposite process. Thus, the maxima or minima observed in compressibility curves reflect changes in the orientation or aggregation state of the molecules within the monomolecular layer. These changes can also be interpreted as first- or second-order phase transitions in the compression isotherms. Generally, the maximum of *K* indicates a configurational transition in the structure of the monomolecular layer [35,41,42].

**Figure 10:**
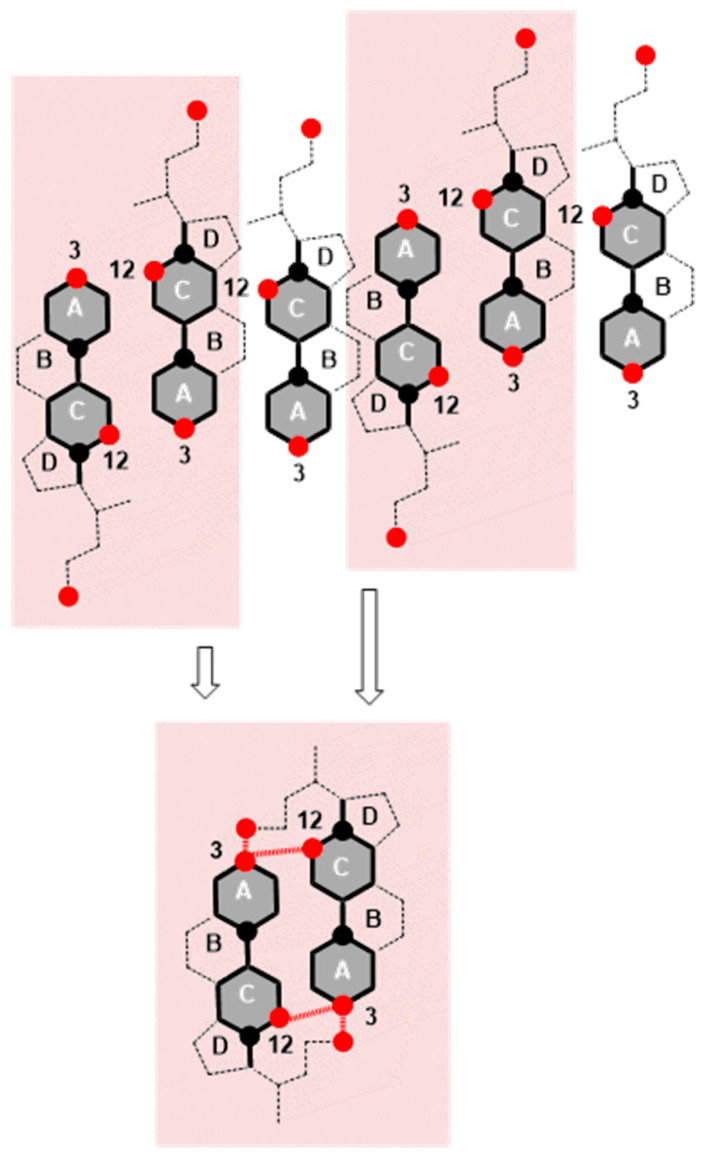
Deoxycholic acids are represented by 2D projections in the plane of the molecular graph of the steroid skeleton; the black circle corresponds to the angular methyl groups C18 and C19, while the red circle corresponds to the OH groups from C3 and C12 carbons and the C24 carboxyl group from the side chain. Hydrogen bonds are formed at the molecular surface from 145 Å to 200 Å during compression (reduction in the surface area) of the monomolecular layer between the suitably oriented deoxycholic acids (facing each other with the C12 side of the steroid skeleton). If one deoxycholic acid is oriented with the C7 lateral side toward the C12 lateral side of another bile acid, then the maximum number of hydrogen bonds is not formed.

In the compression isotherm of deoxycholic acid (Figure 3, tangents), $-{\left(\partial \pi /\partial A\right)}_{T}$ has a maximum value (−tan_max_) in the interval from 120 Å^2^/molecule to 145 Å^2^/molecule and a significantly smaller value in the interval of (145–200) Å^2^/molecule, and the same applies to the compressibility modulus *K*. Probably in the compression isotherm interval of (145–200) Å^2^/molecule, hydrogen bonds are formed between suitably oriented deoxycholic acids (Figure 10 and Appendix A), i.e., the surface of the monomolecular layer shows little resistance when reduced (analogous to the compression of gas in a cylinder).

**Figure 11:**
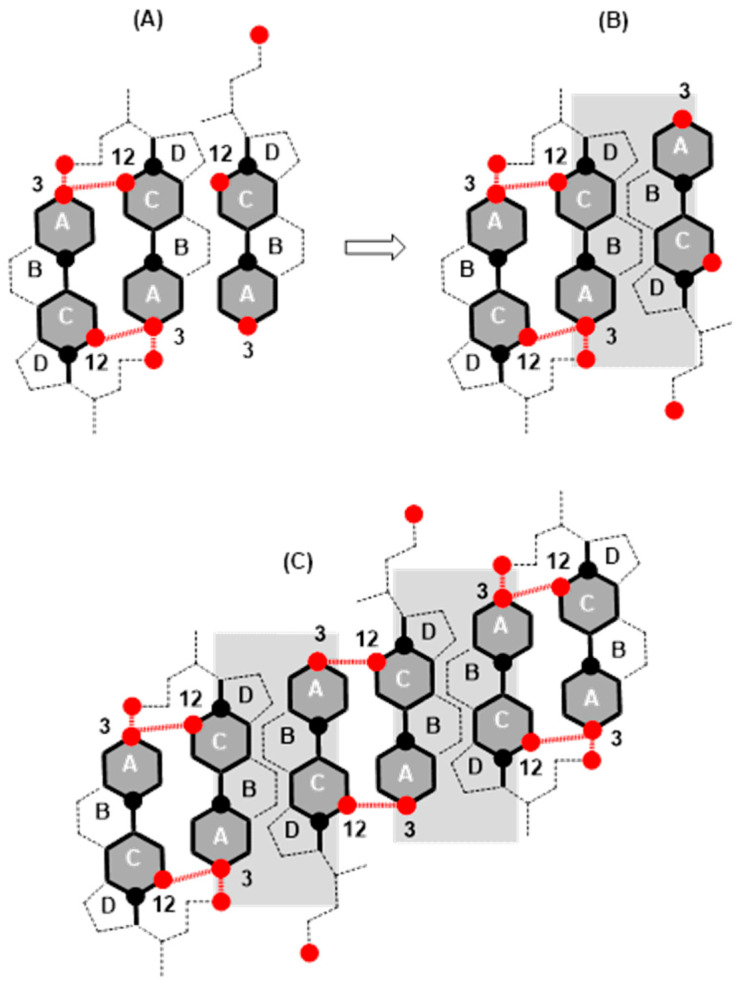
In the structural unit (**A**), one deoxycholic acid is reorientated so that the hydrophobic C7 lateral sides are in mutual contact (**B**), and the surface structural units (**B**) with free C12 OH bonds are further associated with each other through hydrogen bonds (**C**).

In the area with the maximum $-{\left(\partial \pi /\partial A\right)}_{T}$ (Figure 3, −tan_max_), there is a reorientation of deoxycholic acid molecules with their C7 lateral sides (lateral hydrophobic side, i.e., the hydrophobic surface formed by α and β equatorial hydrogen atoms on the side where the C7 carbon of the steroid skeleton is or where cholic acid has an axial OH group; Figure 1) facing the C12 lateral sides of other deoxycholic acid molecules. Since in the 2D surface (monomolecular film) here the deoxycholic acid molecule must turn by 180°, this then represents resistance to the compression of the monolayer film (Figure 11A,B). After reorientation, the hydrophobic C7 lateral sides are in contact with each other in some bile acids, whereby such surface structural units have free C12 lateral OH bonds (Figure 11B). Surface structural units with free C12 OH bonds can be further connected by hydrogen bonds (Figure 11C).

The interaction energy between molecules in a monomolecular film can be calculated using the compression energy experienced by the surface film during compression. This energy is typically in the order of zJ (1 zJ = 10^−21^ J). It is calculated as the product of the surface pressure (*π*, mN/m or mJ/m^2^) and the molecular area (*A*, m^2^ per molecule) associated with the specified surface pressure, assuming constant temperature [36,43,44]:

(3) $$E_{T}=\pi A$$

The interaction energy is usually calculated for each region of the compression isotherm where the slope of the isotherm is constant; the lowest value of the molecular surface is considered for the given region, which then corresponds to the highest value of the surface pressure in the given region of the compression isotherm. Suppose the hypothetical structure of the molecular (surface) aggregate is defined in the given region of the compression isotherm. In this case, the interaction energy (3) represents the energy of forming the hypothetical surface structure. Therefore, the energy of formation of the structural unit (A) (Figure 11), area II of the compression isotherm (145 Å^2^/molecule, Figure 3), is *E_T_*(A) = 10.15 zJ/molecule (energy change due to the formation of hydrogen bonds between deoxycholic acids during the formation of the surface aggregate (A)). The interaction energy of surface aggregate formation (C) at 120 Å/molecule (Figure 3 and Figure 11) is *E_T_*(C) = 30.00 zJ/molecule. This interaction energy originates from the hydrophobic interaction between the C7 lateral sides of the steroid skeletons (B) and the newly formed hydrogen bonds (C) (Figure 11). Let us assume the approximation that there are two orientations in deoxycholic acids according to the formation of intermolecular hydrogen bonds between the steroid skeletons: favorable and non-favorable orientations. Therefore, we can assume (approximately) that half of the hydrogen bonds (up to region III, Figure 3) occur during the formation of the structural unit (A). In contrast, the other half occur after the reorientation of the steroid skeleton during the formation of the surface aggregate (C). It follows that the hydrophobic interaction energy is

(4) $$E_{T}\left(HB\right)=E_{T}\left(C\right)-2E_{T}\left(A\right)$$

Thus, *E_T_*(*HB*) for deoxycholic acid is 9.70 zJ/molecule. The value in the literature of *E_T_*(*HB*) for the same bile acid is 11.90 zJ/molecule, while that for 5*β*-cholanoic acid is 9.50 zJ/molecule [36].

**Figure 12:**
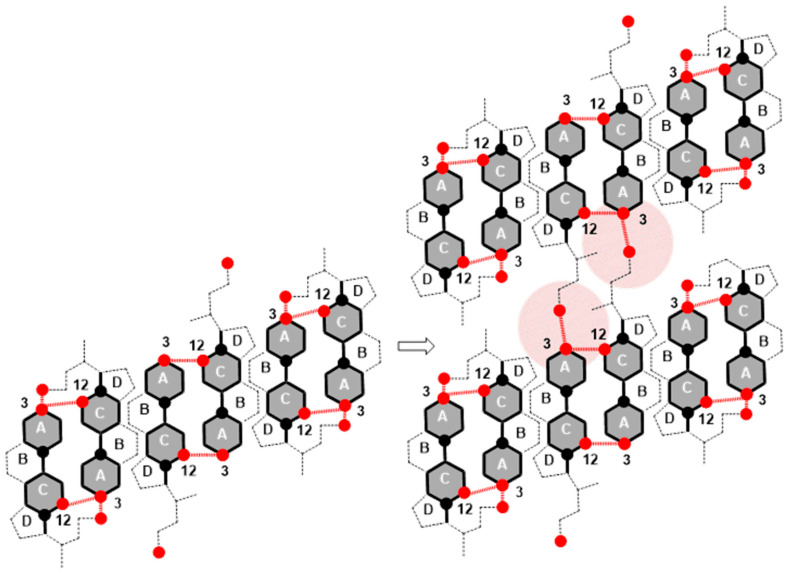
Association of surface structural units via free C24 carboxyl and C3 OH groups of the steroid skeleton.

Once the maximum value of $-{\left(\partial \pi /\partial A\right)}_{T}$ (Figure 3, −tan_max_) is achieved, the value of $-{\left(\partial \pi /\partial A\right)}_{T}$ decreases, i.e., the resistance during compression of the monomolecular layer decreases. Where the molecular surface is in the interval from 80 Å^2^ to 120 Å^2^ in the compression isotherm, the mutual orientations of deoxycholic acids probably no longer occur, but structures like (C) (Figure 11) build mutual hydrogen bonds where free C24 carboxyl functions and C3 OH groups participate (Figure 12).

If the molecular surface is in the interval less than 80 Å^2^ (area IV, Figure 3), then $-{\left(\partial \pi /\partial A\right)}_{T}=0$, which means that first-order phase transitions occur. Deoxycholic acids from the tightly bound surface liquid phase (liquid-condensed phase) with a decreased molecular surface are squeezed out and form a macroscopic solid phase.

**Figure 13:**
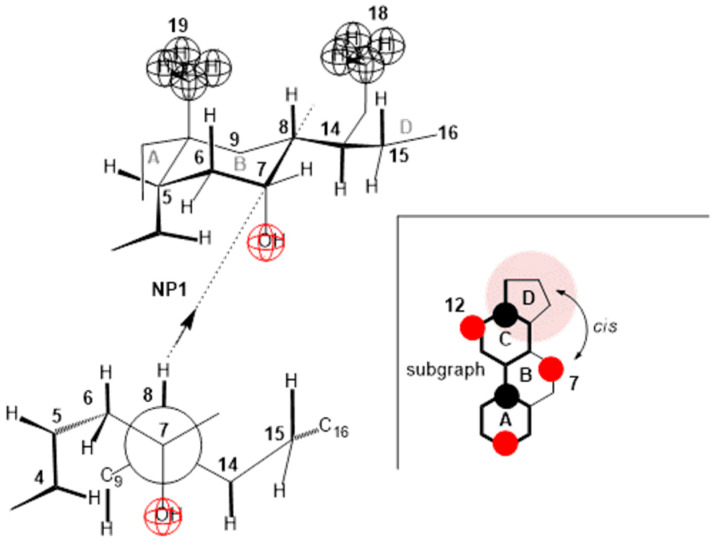
In cholic acid, the α-axial C7 OH group is synaxial with C9 and C14 methine hydrogens, as well as with the C4 methylene group. All these groups screen the approach to the C7 OH group when building a hydrogen bond with another steroid OH group. This also complicates the cis positions of the D ring and the C7 OH group. On the contrary, the C12 OH group does not have a ring that would be in the cis position.

Compared to deoxycholic acid, cholic acid has a C7 α-axial OH group, which, unlike the C12 α-axial OH group, is sterically screened with the D ring of the steroid skeleton, since the C7 OH group and the D ring have a mutual cis orientation in relation to the main subgraph of the steroid skeleton (Figure 13). In addition, the α-axial C7 OH group is in 1,3-synaxial interactions with C9 and C14 methine hydrogens, as well as with the C4 methylene group, which additionally screens this OH group (Figure 13), i.e., it hinders the approach of some other bile acid with a C3 or C12 OH group for the formation of hydrogen bonds [4,45]. Therefore, in terms of the association due to compression in the monomolecular layer, the C7 lateral side of cholic acid behaves like the C7 lateral side of deoxycholic acid. The C7 α-axial OH group of cholic acid, on the α side of the steroid skeleton, can form hydrogen bonds with small molecules, such as water molecules. During compression of the monomolecular layer of cholic acid, the same processes as in the compression of deoxycholic acid probably occur (Figure 10, Figure 11 and Figure 12). However, the compression isotherm of cholic acid with distinct features (Figure 4) compared to the compression isotherm of deoxycholic acid (Figure 3) is a consequence of the solubility of cholic acid in water [13]. What results is that after the collapse point (cp), with the compression of the monolayer, in addition to simultaneous first-order phase transformation, cholic acid is squeezed into the aqueous solution, so even after cp, the slope of the compression curve’s tangent is not zero, i.e., $-{\left(\partial \pi /\partial A\right)}_{T}\neq 0$. Interaction energies cannot be calculated for cholic acid, due to the solubility of C in aqueous solution [36].

**Figure 14:**
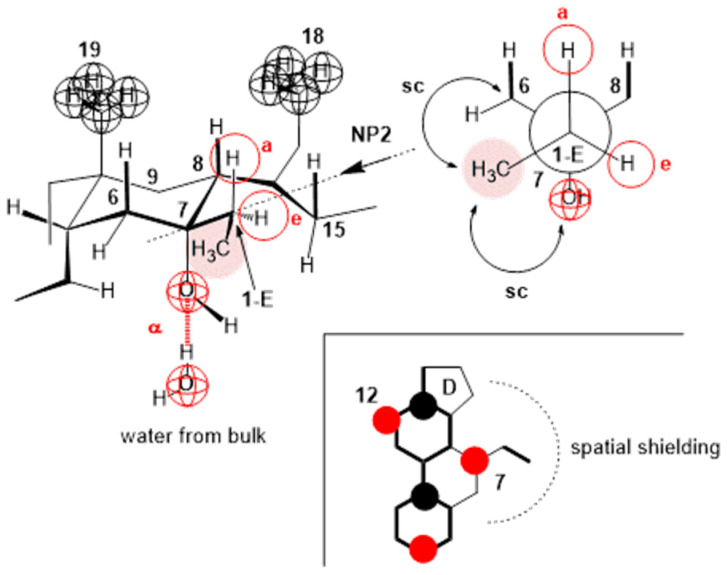
In the 7-EC derivative of cholic acid, the methyl group from the 7β-ethyl group additionally screens the approach to the C7 OH group; it is possible to build a hydrogen bond with water molecules only on the α side of the steroid skeleton, and the position of the terminal methyl group in Newman’s projection formula NP2 is the position in which the methyl group suffers the least steric strain. The molecular graph shows that the methyl group in the orientation from NP2 sterically screens the approach to the C7 OH group from the C7 lateral side of the steroid skeleton.

The compression isotherm of 7-EC (Figure 5 (compression isotherm A)) above 140 Å^2^ (cp) of the molecular surface shows similar characteristics to the compression isotherm of deoxycholic acid. However, when the monomolecular film is compressed, and the molecular surface is in the interval less than 140 Å^2^, the compression isotherm shows similar characteristics to the compression isotherm of cholic acid (Figure 4). Namely, with the reduction in the molecular surface after cp, there is a partial dissolution of 7-EC in the aqueous phase. Figure 14 shows the partial conformation of the steroid skeleton of the 7-EC derivative with the 7β-ethyl group, where the terminal methyl group can occupy the optimal orientation (spatial position). In addition to the indicated spatial position, positions a and e theoretically are also possible; however, in position a, the methyl group is in synaxial interactions [46] with C6, C8, and C15 axial hydrogens, which creates steric strain. Similarly, position e (orientation toward the D ring) creates a syn-pentane interaction and thereby steric strain [47]; therefore, positions a and e are unsuitable. The methyl group in the current (optimal) position (Figure 14) in Newman’s projection formula (NP2) additionally sterically screens the C7 axial OH group from the C7 lateral side of the steroid skeleton. However, access to water molecules from the internal aqueous phase (substrate) is possible from the α side of the steroid skeleton, similarly to cholic acid, increasing the solubility of 7-EC derivatives in the aqueous substrate.

**Figure 15:**
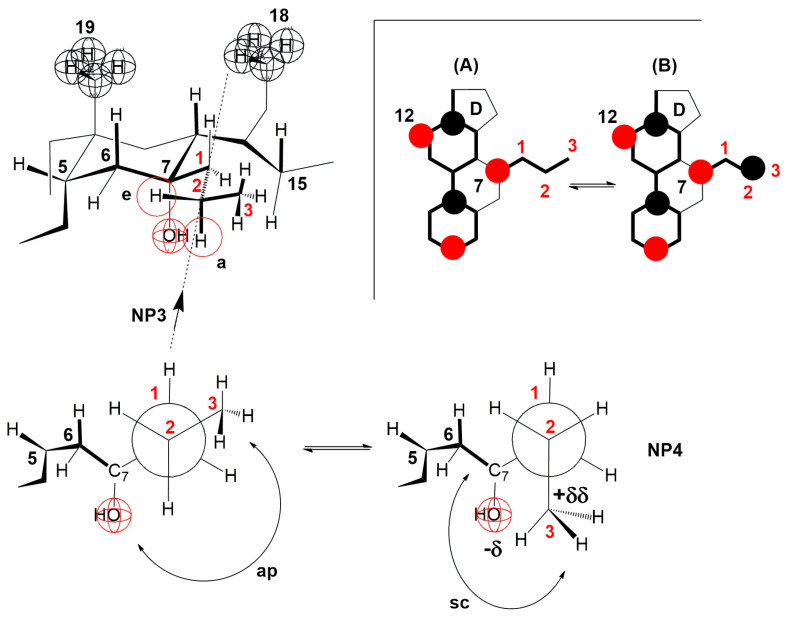
In the 7-PC derivative of cholic acid, the terminal methyl group of the 7β-propyl group, in addition to the antiperiplanar (ap) position in NP3, can also occupy the synclinal (sc) position in NP4, where the dipole-induced dipole interaction partially compensates the steric repulsive interaction with the C7 axial OH group. The methyl group toward NP4 hinders the approach of the water molecule from the interior of the aqueous solution to the C7 axial OH group on the α side of the steroid skeleton. (A) and (B) are representations of the molecular graph for NP3 and NP4, respectively.

The compression isotherm of the 7-PC derivative is slightly shifted to the right compared to the compression isotherm of the 7-EP derivative, probably due to the larger molecular surface of the propyl group. However, the compression isotherm of 7-PC (Figure 5B) completely loses the characteristics of the compression isotherm of cholic acid (Figure 4), i.e., after the collapse point (cp), the tested bile acid is not soluble in the aqueous phase. However, a first-order phase change occurs, i.e., displacement of the 7-PC derivative from the monomolecular film into a water-insoluble solid phase. Although the compression isotherm of 7-PC relative to the compression isotherm of 7-EC shifts slightly to the right, the collapse points coincide (140 Å^2^/molecule, Figure 5). This can be explained by the balance between the conformation in which the terminal methyl group of the 7β-propyl group is in the antiperiplanar (ap) position to the C7 axial OH group (NP3, Figure 15) and the conformation in which the terminal methyl group of the 7β-propyl group is in the synclinal position (sc) to the C7 axial OH group (NP4, Figure 15). In the conformation corresponding to Newman’s projection formula NP4, a particular steric strain exists between the C7 axial OH and terminal methyl groups. However, this steric strain is partially compensated by the dipole-induced dipole attractive interaction (Figure 15). In the conformation corresponding to Newman’s projection formula NP4, the terminal methyl group of the 7β-propyl group hinders the access of the water molecule from the interior of the water substrate to the C7 axial OH group (the access of the water molecule from the α side of the steroid skeleton is hindered), i.e., the C7 OH group is sterically masked (screened) to form a hydrogen bond, which is why when the monomolecular film is compressed after the collapse point, the 7-PC derivative is not extruded into the aqueous substrate (i.e., it does not dissolve in the aqueous phase). Otherwise, both conformations of the 7β-propyl chain (NP3 and NP4) from the C7 lateral side spatially screen the C7 axial OH group (Figure 15 molecular graphs: (A) and (B)). Although the C7 OH group is present due to the 7β-propyl group, the C7 lateral side of the 7-PC derivative is hydrophobic, corresponding to the orientation changes from Figure 10, Figure 11 and Figure 12 during the compression of the monomolecular film.

The terminal methyl group of the C7 propyl group cannot occupy the e position (partial conformation of the steroid skeleton, Figure 15), because, in that case, a syn-pentane repulsive interaction occurs with the C6 equatorial hydrogen, which cannot be realized at room temperature [40]. Otherwise, if the terminal methyl group of the C7 propyl group occupies the e position, then the access of water molecules from the aqueous substrate to the C7 axial OH group would be free from the α side of the steroid skeleton. The 7-PC derivative would partially dissolve in the aqueous phase after the collapse point, i.e., the compression isotherm would resemble that of the 7-EP derivative of cholic acid.

**Figure 16:**
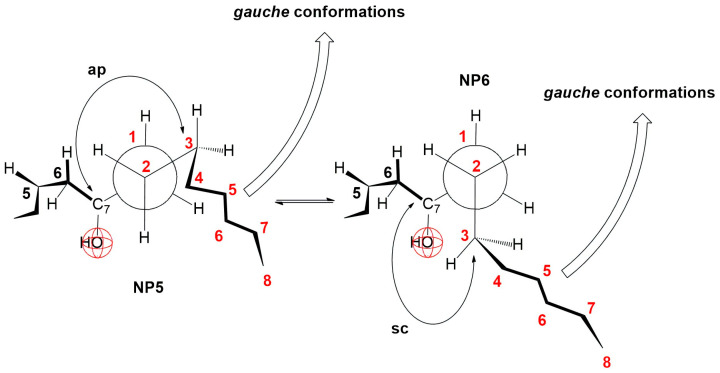
Fragmentary conformations of 7-BC and 7-OC derivatives in partial Newman projection formulas.

In the derivatives 7-BC and 7-OC, the compression isotherms shift toward higher values of the surface pressure and molecular surface (Figure 5), where the isotherms have a similar course to the compression isotherm of deoxycholic acid (Figure 3). Both derivatives have probable partial conformations analogous to the conformations represented by Newman’s projection formulas NP3 and NP4 (Figure 15 and Figure 16). With both alkyl derivatives of cholic acid, when the monomolecular layer is compressed, the orientational transformations shown in Figure 10, Figure 11 and Figure 12 are likely. In addition, when the molecular surface is reduced, i.e., upon compression of the monolayer film, conformational changes of the C7 side chain are possible. However, after the collapse point, the compression isotherm becomes horizontal (Figure 5C,D); this means that the derivatives 7-BC and 7-OC do not dissolve in the aqueous substrate, which is due to the access of water molecules to the α side of the steroid skeleton in the vicinity of the C7 α-axial OH group being sterically hindered. This is possible if at least one methylene group from the C7 alkyl chain is in the sc position toward the C7 OH group (NP6, Figure 16). At the same time, the rest of the alkyl chain can take gauche conformations that occupy a smaller surface when compacting the monomolecular film.

The energy of the hydrophobic interaction (*E_T_*(*HB*), as shown in Table 2), between the 7β-alkyl derivatives in the monomolecular layer increases with the length of the alkyl chain. However, *E_T_*(*HB*) does not change linearly with the chain length. This observation supports the conformational equilibrium illustrated in Figure 15 and Figure 16, indicating that the octyl chain tends to adopt gauche conformations and positions itself closer to the angular methyl groups of the steroid skeleton. Consequently, the octyl chain contributes less to hydrophobic interactions with the C7 lateral side of the steroid skeleton during the formation of aggregates in the surface layer of bile acids than would be expected based solely on the number of carbon atoms in the hydrocarbon chain, i.e., when the two octyl chains enter into a hydrophobic interaction with each other along the entire length of the chain.

**Figure 17:**
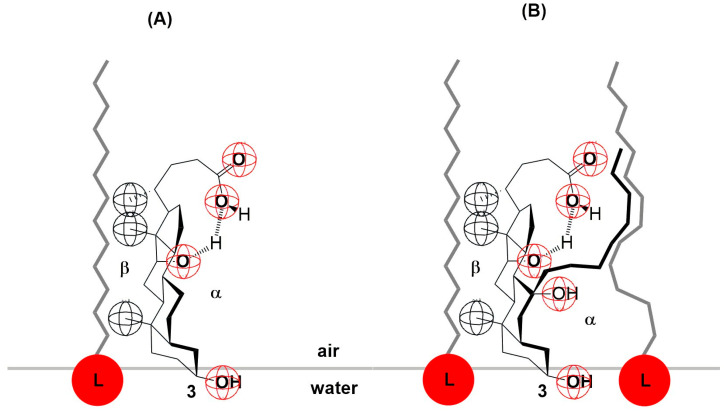
Deoxycholic acid (**A**) and 7-OC (**B**) interaction with lecithin (L) at the interface.

In the deoxycholic acid–lecithin and 7-OC–lecithin binary mixtures in a monomolecular film, there are synergistic interactions between different molecules and similar orientations of bile acids. With deoxycholic acid, line III cuts the abscissa at 75 Å^2^/molecule (Figure 6), while in 7-OC, line III cuts the abscissa at 80 Å^2^/molecule (Figure 9B). These molecular surfaces correspond to the cross section of the steroid skeleton, which means that only the C3 pseudoaxial OH group is immersed in the aqueous solution, while the hydrophobic surface of the steroid skeleton is next to the hydrophobic surface of the alkyl chain of the acyl group of lecithin (Figure 17). In the 7-OC derivative, the C7 α-axial OH group is sterically shielded by the C7 octyl chain; therefore, for steric reasons, the formation of a hydrogen bond between the C7 α-axial OH group and water molecules from the aqueous phase is hindered (the water solubility of the steroid skeleton on the C7 lateral side reduces). Further, the length of the octyl chain allows both gauche and possibly synperiplanar conformations (after the first three C atoms of the alkyl chain) without creating a steric strain with the steroid skeleton [40]. Therefore, hydrophobic interactions between the C7 octyl chain and some lecithin molecules are possible (Figure 17). In 7-BC derivatives, there are also synergistic interactions in the binary mixture with lecithin in the monomolecular layer (Figure 9A); however, the insufficient length of the C7 butyl chain results in the absence of the orientation of 7-BC in which only the C3 OH group is immersed in the aqueous phase, i.e., the butyl chain is unable to enter into hydrophobic interactions with lecithin (line III intersects the abscissa at 160 Å^2^).

The inverse capacity of the sodium salts of bile acids in lecithin solubilization (denoted as 1/x_L_) indicates the number of moles of sodium salts of bile acids required to solubilize 1 mole of lecithin [32] under equilibrium conditions in the submicellar region [48]. As observed in Table 3, to solubilize 1 mole of lecithin, we require at least sodium salts of deoxycholic acid and 7-OC. This finding confirms the interactions between the tested bile acids and lecithin in the monomolecular film, as illustrated in Figure 17. Furthermore, the less sterically hindered the C7 α-axial hydroxyl group of the steroid skeleton, the greater the amount of sodium salts of bile acid needed to solubilize 1 mole of lecithin.

A comparison of the association of particles in the monomolecular layer and the interior of the aqueous solution (bulk) shows that the common feature is the structural diversity of the aggregates, which gradually changes with a decrease in the molecular surface of the monolayer (surface concentration and surface pressure increase), i.e., with an increase in the concentration of the surfactant (bile acid salts) in the aqueous solution: starting from premicellar aggregates, primary micelles (mainly hydrophobic interactions between micellar building units), and secondary micelles (mainly hydrogen bonds between primary micelles). However, both primary and secondary micelles exhibit polydispersity in terms of the aggregation number, confirming a gradual association (stepwise aggregation) [14,15,49,50,51,52,53,54,55,56,57]. During the formation of a bile acid salt’s primary micelles at room temperature, the main driving force is entropic [8,9,10], and it follows that water molecules from the hydration layer above the hydrophobic surface of the steroid skeleton enter the interior of the bulk solution, where they have higher entropy (formal process)—the hydrophobic effect [58]. Therefore, during the dehydration of the hydrophobic surface of the steroid skeleton, the entropy of the system increases. In order to prevent re-hydration, at and above the critical micellar concentration (CMC), bile acid anions join the hydrophobic surfaces of the steroid skeleton into micelles. (Of course, complete protection of the hydrophobic surfaces in the micellar state is not possible, i.e., even in the micellar state, the hydrophobic surfaces are partially hydrated.) In an ideal monomolecular layer, the hydrophobic surfaces of the steroid skeletons of bile acids are in the air, i.e., they are in a dehydrated state. The joining of hydrophobic surfaces occurs when the molecular surface (in the monomolecular layer) is reduced when the steroid skeletons come close to each other. Dipole-induced dipole and induced dipole-induced dipole electrostatic interactions are formed—hydrophobic interactions [58]. Generally, the larger the hydrophobic surface area between which the interactions occur in a monomolecular film, the higher the hydrophobic interaction energy (*E_T_*(*HB*) increases with the number of C atoms in the C7 alkyl chain (Table 2)). At the same time, with bile acid salts, the larger the hydrophobic surface of the steroid skeleton, the greater the entropic effect in the formation of micelles (a greater number of water molecules participate in the hydration of the hydrophobic surface). In other words, the value of the critical micellar concentration decreases, i.e., the tendency toward self-association increases (the CMC increases in the sequence 7-OC < 7-BC < 7-PC, i.e., the tendency toward the formation of micelles decreases [39]).

## 4. Materials and Methods

The synthesis and chemical characterizations of 7-EC, 7-PC, 7-BC, and 7-OC derivatives (Figure 2) have been published previously [39,40]. Cholic acid (Sigma, Auckland, New Zealand; purity ≥ 99%), deoxycholic acid (Sigma, Auckland, New Zealand; purity ≥ 99%), and lecithin (L-α-phosphatidylcholine: egg yolk; Sigma-Aldrich Burlington, USA; purity ≥ 99%) were used as received. All bile acids were transformed into sodium salts following a known procedure [1].

Langmuir trough experiments [36]: Bile acids (or bile acid–lecithin mixtures) were spread from chloroform solutions onto aqueous subphases (pH 2 (Britton–Robinson buffer)* and 3M NaCl [13]) using a Kibron *μ*TroughXS (Kibron Inc., Helsinki, Finland) Langmuir trough. Chloroform was used to prepare bile acid (or all amphiphile molecule) solutions at approximately 0.1 mg/mL. To create a monomolecular film, 40–60 µL of the solutions was applied to spread about 9 moles of molecules on an aqueous surface, resulting in a surface density of 200–300 Å^2^/molecule. The spreading solvent (chloroform) was allowed to evaporate for 5 min before surface compression of the monolayers. A small-diameter (0.51 mm) specialized metal alloy wire was used to measure the surface pressure with a resolution of 0.2 mg and a sensitivity better than 0.01 mN/m. The bile acid (or all amphiphile molecule) films were compressed at a speed of 6.5 mm^2^/min, with an area measurement inaccuracy of less than 0.5%. The experiments were conducted at a constant temperature of 298.15 ± 0.1 K, controlled with a thermostat and the Kibron temperature control plate. Each experiment was repeated at least three times, and the surface pressure–molecular area curves were reproducible to within 1%. *A Britton–Robinson buffer, consisting of a solution of 0.04 M acetic acid, 0.04 M phosphoric acid, and 0.04 M boric acid, was used in all the experiments and its pH was adjusted to 2 by adding adequate amounts of 0.2 M sodium hydroxide.

Solubilization of lecithin: Our modified version of the equilibrium submicellar solubilization process of lecithin with Na salts of bile acids was applied [32].

## 5. Conclusions

The compression isotherm of deoxycholic acid differs from that of cholic acid in a few key ways. For deoxycholic acid, after reaching the collapse point during the compression of the monomolecular film, there is a region where the surface pressure remains constant. In contrast, the surface pressure changes after the collapse point with cholic acid. This difference can be explained by the solubility of cholic acid in the aqueous substrate.

In the case of 7-EC derivatives, the compression isotherm resembles that of cholic acid, while the compression isotherms of the 7-PC, 7-BC, and 7-OC derivatives display characteristics similar to those of deoxycholic acid. This phenomenon is related to the steric shielding of the C7 α-axial OH group by the C7 alkyl chain, which makes it difficult for water molecules to approach from the α side of the steroid skeleton (the OH group).

There are synergistic interactions in the binary mixture of deoxycholic acid and lecithin and the mixture of 7-OC and lecithin within the monomolecular film. In these mixtures, the bile acids position themselves so that only the C3 pseudoaxial OH group is immersed in the aqueous phase.

These insights could be relevant for designing bile acid-based carriers for membrane drug delivery, where monolayer stability and orientation are critical.

## Figures and Tables

**Table 1:** Parameters of collapse points at 298.15 K on the substrate of an acidic aqueous solution with pH 2 and an NaCl concentration of 3 M.

Title 1	Area per Molecule (Å^2^)	π (mNm^−1^)
D	80.0 ± 0.8	30.0 ± 0.3
C	105.0 ± 1.2	11.8 ± 0.1
7-EC	140.0 ± 1.6	12.0 ± 0.1
7-PC	141.0 ± 1.6	15.9 ± 0.14
7-BC	152.0 ± 1.7	19.8 ± 0.18
7-OC	164.0 ± 1.7	27.6 ± 0.25

**Table 2:** Interaction energies according to Equations (3) and (4) during the formation of structural units (A) and (C), as well as the energy of hydrophobic interactions in the surface monolayer at 298.15 K.

	D	C	7-EC	7-PC	7-BC	7-OC
	zJ/Molecule					
$E_{T}\left(A\right)$	10.15	n.d.	n.d.	5.70	4.32	9.20
$E_{T}\left(C\right)$	30.0			18.5	26.60	39.9
$E_{T}\left(HB\right)$	9.70			7.1	17.96	21.5

Since the method of determining the interaction energy is approximate, the error is not determined [36]; n.d. = not defined due to the solubility of bile acids in the aqueous phase.

**Table 3:** Inverse capacity (1/x_L_) of Na salts of bile acids in the solubilization of lecithin at 298.15 K in the submicellar region (aqueous solution with pH 7.4).

	D	C	7-EC	7-PC	7-BC	7-OC
1/x_L_	0.35 ± 0.03	0.65 ± 0.08	0.58 ± 0.06	0.48 ± 0.05	0.40 ± 0.03	0.32 ± 0.03

**Figure A1:**
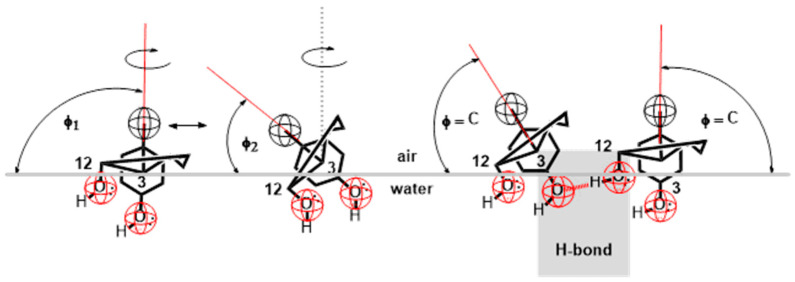
Formation of bile acid associates in the surface monolayer via H-bonding.

## Data Availability

All the relevant data are provided within the paper.

## Abbreviations

The following abbreviations are used in this manuscript:

D	Deoxycholic acid
C	Cholic acid
7-EC	7β-Ethylcholic acid
7-PC	7β-Propylcholic acid
7-BC	7β-Butylcholic acid
7-OC	7β-Octylcholic acid
cp	Collapse point of the surface film
sc	Synclinal
ap	Antiperiplanar
NP	Newman projection

## Appendix A

In the monomolecular surface layer of bile acids, the axis aligned with the axial bond of the angular methyl group can form various angles with the air–water interface (Φ). This suggests that there may be fluctuations in the values of Φ. Additionally, at each value of Φ, there exists an axis perpendicular to the boundary surface around which the steroid skeleton can rotate (see Figure A1).

During the formation of hydrogen bonds between bile acids, Φ becomes constant (Φ = C), with values that are optimal for hydrogen bonding. For bile acids, as they form hydrogen bonds in their surface associates, both the axial and pseudoaxial hydroxyl (OH) groups remain submerged in the aqueous phase, where they also engage in hydrogen bonding with water molecules. It is important to note that these hydrogen bonds exist even prior to the association of bile acids; therefore, any changes in the aqueous substrate are generally not depicted.

## References

[1] Roda A., Hofmann A.F., Mysels K.J. (1983). The influence of bile salt structure on self-association in aqueous solutions. J. Biol. Chem..

[2] Madenci D., Egelhaaf U.S. (2010). Self-assembly in aqueous bile salt solutions. Curr. Opin. Colloid Interface Sci..

[3] Hofmann A.F., Roda A. (1984). Physicochemical properities of bile acids and their relationship to biological properties: An overview of the problem. J. Lipid Res..

[4] Poša M. (2014). Heuman indices of hydrophobicity in bile acids and compared with new developed and conventional molecular descriptors. Biochimie.

[5] Kumar D., Poša M. (2023). Linear hydrophobic congeneric groups of bile acid anion derivatives based on the self-association (micellization) process and the phenomenon of enthalpy-entropy compensation. J. Mol. Liq..

[6] Natalini B., Sardella R., Camaioni E., Macchiarulo A., Gioiello A., Carbone G., Pellicciari R. (2009). Derived chromatographic indices as effective tools to study the self-aggregation process of bile acids. J. Pharm. Biomed. Anal..

[7] Natalini B., Sardella R., Gioiello A., Ianni F., Di Michele A., Marinozzia M. (2014). Determination of bile salt critical micellization concentration on the road to drug discovery. J. Pharm. Biomed. Anal..

[8] Garidel P., Hildebrand A. (2005). Thermodynamic properties of association of colloids. J. Therm. Anal. Cal..

[9] Garidel P., Hildebrand A., Neubert R., Blume A. (2000). Thermodynamic characterization of bile salt aggregation as a function of temperature and ionic strength using isotermal titration calorimetry. Langmuir.

[10] Anderson S.L., Rovnyak D., Strein T.G. (2016). Direct measurement of the thermodynamics of chiral recognition in bile salt micelles. Chirality.

[11] Vázquez-Tato M.P., Seijas J.A., Meijide F., Fraga F., de Frutos S., Miragaya J., Trillo J.V., Jover A., Soto V.H., Vázquez Tato J. (2021). Highly Hydrophilic and Lipophilic Derivatives of Bile Salts. Int. J. Mol. Sci..

[12] Kawamura H., Murata Y., Yamaguchi T., Igimi H., Tanaka M., Sugihara G., Kratohvil J.P. (1989). Spin-label studies of bile salt micelles. J. Phys. Chem..

[13] Small D.M., Nair P.P., Kritchevsky D. (1971). The physical chemistry of cholanic acids. The Bile Acids: Chemistry, Physiology and Metabolism.

[14] Meier A.R., Yehl J.B., Eckenroad K.W., Manley G.A., Strein T.G., Rovnyak R. (2018). Stepwise aggregation of cholate and deoxycholate dictates the formation and loss of surface-available chirally selective binding sites. Langmuir.

[15] Rovnyak D., He J., Kong S., Eckenroad K.W., Manley G.A., Geffert R.M., Krout M.R., Strein T.G. (2023). Determining sequential micellization steps of bile salts with multi-CMC modeling. J. Colloid. Interface. Sci..

[16] Pártai L.B., Sega M., Jedlovszky P. (2007). Morphology of bile salts micelles as studied by computer simulation methods. Langmuir.

[17] Haustein M., Schiller P., Wahab M., Mogel H.J. (2014). Computer simulations of the formation of bile salt micelles and bile salt/DPPC mixed micelles in aqueous solutions. J. Solution Chem..

[18] Poša M. (2023). Self-association of the anion of 7-oxodeoxycholic acid (bile salt): How the secondary micelles are formed. Int. J. Mol. Sci..

[19] Mikov M., Fawcett J.P. (2007). Bile Acids.

[20] di Gregorio M.C., Cautela J., Galantini L. (2021). Physiology and physical chemistry of bile acids. Int. J. Mol. Sci..

[21] Rub M.A., Azum N., Khan F., Asiri A.M. (2018). Aggregation of sodium salt of ibuprofen and sodium taurocholate mixture in different media: A tensiometry and fluorometry study. J. Chem. Thermodyn..

[22] Kumar D., Farakaš Agatić Z., Popović K., Poša M. (2024). Binary mixed micelles of hexadecyltrimethylammonium bromide—Sodium deoxycholate and dodecyltrimethylammonium bromide—Sodium deoxycholate: Thermodynamic stabilization and mixed micelle’s solubilization capacity of daidzein (isoflavonoid). Ind. Eng. Chem. Res..

[23] Enache M., Toader A.M., Neacsu V., Ionita G., Enache M.I. (2017). Spectroscopic investigation of the interaction of the anticancer drug mitoxantrone with sodium taurodeoxycholate (NaTDC) and sodium taurocholate (NaTC) bile salts. Molecules.

[24] Toader A.M., Dascalu I., Neacsu E.I., Enache M. (2023). Binding interactions of actinomycin D anticancer drug with bile salts micelles. J. Serb. Chem. Soc..

[25] Wiedmann T.S., Kamel L. (2002). Examination of the solubilization of drugs by bile salt micelles. J. Pharm. Sci..

[26] Maya S.A., Alam M.M., Khan J.M., Anis-Ul-Haque K.M., Rana S., Hasan K., Posa M., Kumar D., Rahman M.M., Hoque M.A. (2025). The aggregation behaviour of tetradecyltrimethylammonium bromide in aqueous solution of an antidiabetic drug at variable temperatures: Influences of dihydroxy organic compounds and temperature. Colloids Surf. A.

[27] Tepavčević V., Farkaš Agatić Z., Pilipović A., Puača G., Poša M. (2025). Effect ofβ-Cyclodextrin on the Aggregation Behavior of Sodium Deoxycholate and Sodium Cholate in Aqueous Solution. Molecules.

[28] Al-Salami H., Butt G., Tucker I.G., Mikov M. (2008). Influence of the semisynthetic bile acid MKC on the ileal permeation of gliclazide in vitro in healthy and diabetic rats treated with probiotics. Methods Find. Exp. Clin. Pharmacol..

[29] Yang L., Zhang H., Mikov M., Tucker I.G. (2009). Physicochemical and biological characterization of monoketocholic acid, a novel permeability enhancer. Molecular Pharmaceutics.

[30] Yang L., Fawcett J.P., Østergaard J., Zhang H., Tucker I.G. (2012). Mechanistic studies of the effect of bile salts on rhodamine 123 uptake into RBE4 cells. Molecular Pharmaceutics.

[31] Garidel P., Hildebrand A., Knauf K., Blume A. (2007). Membranolytic activity of bile salts: Influence of biological membrane properities and composition. Molecules.

[32] Poša M., Kuhajda K. (2010). Hydrophobiciti and haemolytic potential of oxo derivatives of cholic, deoxycholic and chenodeoxycholic acids. Steroids.

[33] Blume A. (2018). Lipids at the air–water interface. ChemTexts.

[34] Giner-Casares J.J., Brezesinski G., Möhwald H. (2014). Langmuir monolayers as unique physical models. Curr. Opin. Colloid Interface. Sci..

[35] Messina P.V., Prieto G., Ruso J.M., Fernandez-Leyes M.D., Schulz P.C., Sarmiento F. (2010). Thermodynamic and elastic fluctuation analysis of langmuir mixed monolayers composed by dehydrocholic acid (HDHC) and didodecyldimethylammonium bromide (DDAB). Colloids Surf. Biointerfaces.

[36] Szekeres M., Viskolcz B., Poša M., Csanádi J., Škorić D., Illés E., Tóth Y.I., Tombácz E. (2014). The effect of hydroxyl moieties and their oxosubstitution on bile acid association studied in floating monolayers. Sci. World J..

[37] Poša M., Bjedov S., Tepavčević V., Mikulić M., Sakač M. (2020). Physicochemical characterization of novel 3-carboxymethyl-bile salts, as permeability and solubility enhancers. J. Mol. Liq..

[38] Rojewska M., Smułek W., Grzywaczyk A., Kaczorek E., Prochaska K. (2023). Study of Interactions between saponin biosurfactant and model biological membranes: Phospholipid monolayers and liposomes. Molecules.

[39] Poša M., Pilipović A., Bjedov S., Obradović S., Tepavčević V., Sakač M. (2016). Parameters of micellization and hydrophobicity of sodium salts of 7- buthyl (butylidene) and 7-octyl (octylidene) derivatives of the cholic and the deoxycholic acid in a water solution: Pattern recognition—Linear hydrophobic congeneric groups. J. Mol. Liq..

[40] Kumar D., Poša M. (2024). Thermodynamics of micelle formation of selected homologous 7-alkyl derivatives of Na-cholate in aqueous solution: Steroid skeleton and the alkyl chain conformation. Int. J. Mol. Sci..

[41] Galvez-Ruiz M.J., Cabrerizo-Vılchez M.A. (1991). Structural and stability analysis of monolayers of some bile acids at the airaqueous solution interface. Colloids Surf..

[42] Ekwall P., Ekholm R., Norman A. (1957). Surface balance studies of bile acid monolayers, I. Cholanic and Glycocholanic monolayers. Acta Chem. Scand..

[43] Visser J. (1972). On Hamaker constants: A comparison between Hamaker constants and Lifshitz-van der Waals constants. Adv. Colloid Interface Sci..

[44] Dynarowicz P., Jawień W., Miñones Trillo J., Vila Romeu N., Varela Sanchez-Caballero C., Iribarnegaray Jado E., Conde Mouzo O. (1995). Molecular interaction in mixed spread films at the water air interface. Colloids Surf. B.

[45] Poša M., Sebenji A. (2014). Determination of the number average aggregation numbers of bile salt micelles with a special emphasis on their oxo derivatives—The effect of the steroid skeleton. BBA-Gen. Subjects.

[46] Dragojlovic V. (2015). Conformational analysis of cycloalkanes. ChemTexts.

[47] Carlier P.R., Zhang Y., Slebodnick C., Lo M.M.-C., Williams I.D. (2006). Effect of 2,6-Disubstituted Aryl Groups on Acyclic Conformation: Preference for an Antiperiplanar Orientation of the Geminal and Vicinal Hydrogens. J. Org. Chem..

[48] Baskin R., Frost L.D. (2008). Bile salt-phospholipid aggregation at submicellar concentrations. Colloids Surf. B.

[49] Kauffman J.M., Pellicciari L., Carey M.C. (2005). Interfacial properties of most monofluorinated bile acids deviate markedly from the natural congeners: Studies with the Langmuir-Pockels surface balance. J. Lipid Res..

[50] Bollenbach L., Trutschel M.-L., Gröger S., Garidel P., Mäder K. (2025). Interfacial and self-association behaviour of poloxamer 188 in aqueous solutions. J. Mol. Liq..

[51] Guruge A.G., Warren D.B., Pouton C.W., Chalmers D.K. (2023). Molecular Dynamics Simulation Studies of Bile, Bile Salts, Lipid-Based Drug Formulations, and mRNA−Lipid Nanoparticles: A Review. Mol. Pharm..

[52] Pabois O., Ziolek R.M., Lorenz C.D., Prevost S., Mahmoudi N., Skoda M.W.A., Welbourn R.J.L., Valero M., Harvey R.D., Grundy M.M. (2021). Morphology of bile salts micelles and mixed micelles with lipolysis products, from scattering techniques and atomistic simulations. J. Colloid Interface Sci..

[53] Kabedev A., Hossain S., Hubert M., Larsson P., Bergström C.A.S. (2021). Molecular Dynamics Simulations Reveal Membrane Interactions for Poorly Water-Soluble Drugs: Impact of Bile Solubilization and Drug Aggregation. J. Pharm. Sci..

[54] di Gregorio M.C., Travaglini L., Del Giudice A., Cautela J., Pavel N.V., Galantini L. (2019). Bile Salts: Natural Surfactants and Precursors of a Broad Family of Complex Amphiphiles. Langmuir.

[55] Galantini L., di Gregorio M.C., Gubitosi M., Travaglini L., Vázquez J., Jover A., Meijide F., Soto V.H., Pavel N.V. (2015). Bile Salts and Derivatives: Rigid Unconventional Amphiphiles as Dispersants, Carriers and Superstructure Building Blocks. Curr. Opin. Colloid Interface Sci..

[56] Cautela J., Severoni E., Redondo-Gómez C., di Gregorio M.C., Del Giudice A., Sennato S., Angelini R., D’Abramo M., Schillén K., Galantini L. (2020). Substituent Position and Orientation on the Self-Assembly of Steroid Surfactant Isomers. Colloids Surf. B..

[57] Trillo J.V., Jover A., Galantini L., Tato J.V., Soto V.H., Meijide F., di Gregorio M.C., de Frutos S. (2013). Self-Aggregation Mechanism of a Naphthylamide Cationic Derivative of Cholic Acid. From Fibers to Tubules. RSC Adv..

[58] Privalov P.L., Gill S.J. (1989). The hydrophobic effect: A reappraisal. Pure Appl. Chem..

